# Impact of Renin-Angiotensin System Inhibitors on Disease Characteristics in Patients with Localized Prostate Cancer Treated with Radical Prostatectomy: A European Association of Urology Young Academic Urologists Prostate Cancer Working Group Multi-institutional Study

**DOI:** 10.1016/j.euros.2024.09.005

**Published:** 2024-10-08

**Authors:** Nastasiia Artamonova, Mona Kafka, Laura Faiss, David Avetisyan, Ignacio Puche Sanz, Giulia La Bombarda, Gennaio Iacono, Fabio Zattoni, Eberhard Steiner, Caroline D’Elia, Armin Pycha, Michael Ladurner, Samed Jagodic, Giorgio Gandaglia, Isabel Heidegger

**Affiliations:** a Department of Urology, Medical University Innsbruck, Innsbruck, Austria; b UGC Urología, Hospital Universitario Virgen de las Nieves, Granada, Spain; c Department of Urology, University of Padova, Padova, Italy; d Department of Medicine, University of Padova, Padua, Italy; e Department of Urology, Zentralkankenhaus Bozen, Bozen, Italy; f Department of Urology, University of Tuzla, Tuzla, Bosnia and Herzegovina; g Department of Urology, Urological Research Institute Vita-Salute University and San Raffaele Hospital, Milan, Italy

**Keywords:** Cancer aggressiveness, Prostate cancer, Renin-angiotensin system inhibitor, Real-world evidence

## Abstract

**Background and objective:**

Collagen biosynthesis is intricately involved in the development and progression of solid tumors. Renin-angiotensin system inhibitors (RASi) impede TGF-β-mediated collagen synthesis in tumors by hindering activation of the angiotensin receptor. Our aim was to investigate a potential association between RASi use and the aggressiveness of prostate cancer (PCa).

**Methods:**

We conducted a retrospective multicenter analysis for a cohort of 1250 patients with PCa who underwent radical prostatectomy (RP) between 1990 and 2023 in four European high-volume centers. The study cohort comprised 625 RASi-treated patients and 625 age-matched RASi-naïve patients. Data for various parameters were collected, including age at RP, body mass index (BMI), prostate volume, prostate-specific antigen (PSA), percentage of free PSA, Gleason score (GS) at biopsy and RP, TNM stage, and the rate of biochemical recurrence (BCR). Clinical parameters for patients with and without RASi treatment were documented. Differences between the groups were compared using a Mann-Whitney U test and χ^2^ tests. Survival analyses were performed using the Kaplan-Meier method.

**Key findings and limitations:**

As expected, the RASi group had higher BMI levels than the RASi-naïve group (*p* < 0.001). However, RASi use was not associated with key markers of PCa aggressiveness such as GS upgrading from biopsy to RP (*p* = 0.089), surgical margin status (*p* = 0.109), and lymph node involvement (*p* = 0.33). Moreover, there were no significant differences between the groups in BCR incidence (*p* = 0.258) or the time to BCR (*p* = 0.683).

**Conclusions and clinical implications:**

Our findings indicate that RASi therapy does not have a significant effect on the biological aggressiveness of PCa.

**Patient summary:**

We analyzed data for 1250 patients with prostate cancer and found that the use of a commonly prescribed high blood pressure medication was not associated with a less aggressive form of localized prostate cancer.

## Take Home Message

Inhibitors of the renin-angiotensin system are frequently prescribed for antihypertension therapy and can affect cancer aggressiveness by inhibiting collagen synthesis in several different cancers. However, our study findings for a large cohort of 1250 patients indicate that use of this medication class does not influence the aggressiveness of prostate cancer.

## 1 Introduction

Prostate cancer (PCa) is still the second most common malignancy among males, with more than 1.4 million new cases in 2020 [1]. The main focus of cancer research is currently investigation of the tumor microenvironment and the extracellular matrix (ECM) as increasing evidence indicates that ECM structural alterations, influenced by gene mutations and changes in signaling pathways, are important mechanisms underlying tumor development.

Collagen is a crucial element of the ECM and plays a pivotal role in tumor development and proliferation in various organs. Cancer development induces structural alterations in the ECM, influenced by gene mutations and changes in signaling pathways and collagen distribution. Within the ECM, collagens intertwine to form fibers and establish networks with other matrix components, including hyaluronan [2]. Increases in collagen synthesis and deposition are associated with malignant tumor progression in various organs, including the prostate, breast, pancreas, lung, and skin [3], [4], [5], [6], [7]. A higher risk of metastasis due to extensive collagen deposits can be explained by the role of the collagen fiber network in acting as pathways for migration of metastatic cells [5].

Tension within collagen networks intensifies as collagen and hyaluronan levels increase, affecting the accessibility of blood vessels to chemotherapy and inducing fluid pressure, hypoxia, acidosis, and reduced permeability [3], [8]. Mutations in tumor suppressor genes can alter the collagen architecture, which leads to fibrosis and reinforces cell-collagen loops, thus exacerbating collagen production [2].

TGF-β has a significant influence on collagen synthesis in the tumor microenvironment and is produced by tumor cells or cancer-associated fibroblasts (CAFs). While TGF-β typically inhibits growth and promotes apoptosis in healthy epithelial cells, cancer progression alters its signaling, causing an antagonistic response from CAFs. This leads to oncological progression and metastasis by stimulating cell proliferation and fibroblast secretion, and upregulating matrix proteins such as collagen, hyaluronan, and elastin [8]. In addition, TGF-β hinders ECM degradation by inhibiting matrix metalloproteinases. Consequently, the increase in matrix protein synthesis and decrease in matrix proteinase activity contribute to remodeling of the tumor ECM, potentially leading to a fibrotic response known as desmoplasia, which can be observed in breast, pancreatic, and colon cancers [9]. The fibrotic response in tumors increases tissue stiffness, elevating compressive forces within the tumor. This alters gene expression in cancer cells, enhancing their invasiveness and metastatic potential. Myofibroblast contraction and ECM stiffening further release TGF-β, creating a feedback loop. Intratumoral blood-vessel compression reduces oxygen delivery, promoting immune evasion, malignant progression, metastasis, and reducing therapeutic efficacy [10], [11], [12].

**Fig. 1:**
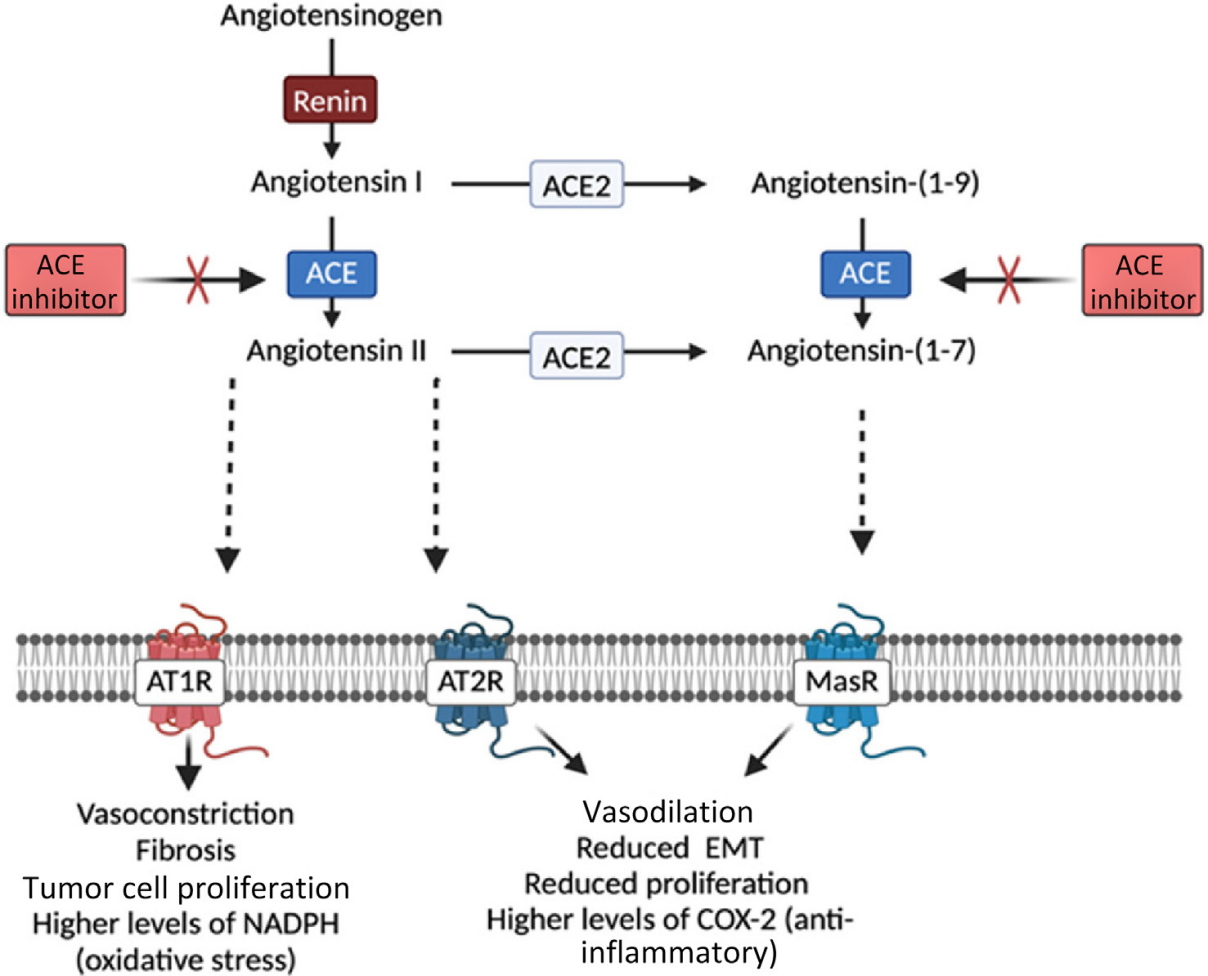
Schematic representation of the impact of the renin-angiotensin system (RAS) and converging signaling cascades within the tumor microenvironment. ACE = angiotensin-converting enzyme; AT1R = angiotensin receptor type 1; AT2R = angiotensin receptor type 2; MasR = MAS receptor.

The renin-angiotensin system (RAS) regulates blood pressure and homeostasis. Renin cleaves angiotensinogen into angiotensin I, which is further converted by angiotensin-converting enzyme (ACE) to angiotensin II, which binds to the receptors AT1R and AT2R, leading to increases in blood pressure via vasoconstriction and water retention (Fig. 1).

RAS inhibitors (RASi), including ACE inhibitors (ACEi) and angiotensin receptor blockers (ARBs), disrupt TGF-β signaling, thereby inhibiting collagen deposition within the ECM. This effect might have a role in preventing metastasis and therefore represents a potential point of action in cancer treatment. Miyajima et al [13] reported a significantly higher 5-yr disease-free survival rate after radical nephrectomy for kidney cancers patients taking RASi (96.8% vs 89.8%; *p* = 0.019). The impact of RASi on PCa remains a topic of debate, as studies have yielded inconsistent results. Some studies suggested a positive influence of RASi on overall survival (OS) [14], [15], [16], some indicated a negative impact of RASi use on the risk of developing PCa [17], [18], while others showed no significant difference between RASi-treated patients and those who were RAS-naïve [19]. Given the heterogeneity of the literature, our aim was to evaluate differences in PCa aggressiveness by RASi use among patients who underwent radical prostatectomy (RP) for organ-confined PCa at one of four European centers between 1990 and 2023.

## 2 Patients and methods

### 2.1 Patient cohort

We conducted a retrospective multicenter study for a cohort of 1250 men who underwent RP for organ-confined PCa between 1990 and 2023 in one of four European centers (Innsbruck, Padova, Granada, Bozen). At the time of RP, 50% of the patients (625/1250) were recieving RASi treatment; 625 age-matched RASi-naïve patients were used as the control group. Patients who had received any adjuvant treatment before RP were excluded from the study. Following parameters were collected and analyzed to evaluate differences between the two groups: age, body mass index (BMI), prostate volume (PV), prostate-specific antigen (PSA), percentage of free PSA (fPSA%), Gleason score (GS) at biopsy and at RP, TNM classification, resection margin status according to the Union for International Cancer Control classification (UICC) [20], biochemical recurrence (BCR), and time to BCR.

### 2.2 Statistical analyses

Data for the two groups were collected in an Excel spreadsheet (version 16.83), and statistical analysis was performed using SPSS (IBM, Armonk, NY, USA). Baseline characteristics were used to describe the population, with the median and range reported for continuous variables. Descriptive statistics such as frequency and cross tables were used to illustrate distributions. To analyze differences between the groups in relation to PCa aggressiveness, the Mann-Whitney U test was used to compare continuous variables and χ^2^ tests to compare results for categorical variables. Recurrence-free intervals were estimated using the Kaplan-Meier method. All statistical tests were two-sided at a significance level of *p* < 0.05.

## 3 Results

### 3.1 Patient characteristics

The study groups were age-matched: median age was 64 yr in the RASi group and 65 yr in the RASi-naïve group. There were no significant differences in PV (*p* = 0.907), PSA (*p* = 0.802), or fPSA% (*p* = 0.848) at diagnosis between the groups, indicating that they were well balanced. However, BMI was significant higher in the RASi group (*p* < 0.001). Baseline patient characteristics are listed in Table 1.

**Table 1:** Baseline characteristics at the time of diagnosis for the two groups

	Overall	Median		Mean (range)		*p* value
	cohort (*n*)	RASi	No RASi	RASi	No RASi	
Age (yr)	1250	64	65	63 (41–77)	65 (46–78)	0.465
BMI (kg/m^2^)	1077	27.28	25.48	28.26 (19.72–39.44)	26.3 (17.17–40.7)	<0.001
PV (ml)	1085	40	40,5	43 (15–135)	47.49 (15–170)	0.931
PSA at Dx (ng/ml)	1216	6	5.9	9.1 (0.5–31.5)	7.2 (0.58–76)	0.959
fPSA (%)	952	11	11	10.65 (0.07-30)	11.25 (0.09-43)	0.917

RASi = renin-angiotensin inhibitor; BMI = body mass index; PV = prostate volume; fPSA = free prostate-specific antigen; Dx = diagnosis.

We categorized patients into three risk groups according to their PSA level before RP. However, there were no significant differences in PSA levels between the RASi group and the control group (*p =* 0.698), indicating no evidence of a less aggressive disease phenotype. (Table 2).

**Table 2:** Characteristics of the two groups at biopsy and RP

Parameter	Patients, *n* (%)		*p* value
	No RASi	RASi	
GS at biopsy			0.172
6	238 (19)	233 (18.6)	
7	219 (17.5)	229 (18.3)	
8	59 (4.7)	75 (6.0)	
9	16 (1.3)	19 (1.5)	
10	1 (0.1)	3 (0.2)	
Unknown	92 (7.4)	66 (5.3)	
GS at RP			0.277
6	125 (10)	116 (9.3)	
7a	258 (20.7)	241 (19.2)	
7b	84 (6.7)	105 (8.4)	
8	38 (3)	50 (4)	
9	48 (3.8)	55 (4.4)	
10	1 (0.1)	3 (0.2)	
Unknown	71 (5.7)	55 (4.4)	
pT stage			0.669
pT2a	114 (9.7)	109 (8.8)	
pT2b	22 (1.8)	18 (1.5)	
pT2c	268 (22.7)	280 (22.5)	
pT3a	121(10.2)	143 (11.5)	
pT3b	54 (4.5)	62 (5)	
pT4	13 (1.1)	9 (0.7)	
pN stage			0.33
pN0	328 (46.5)	338 (47.8)	
pN1	23 (3.2)	17 (2.4)	
Resection margin status			0.109
R0	386 (32)	429 (35.5)	
R1	166 (13.7)	160 (13.3)	
Rx	40 (3.3)	27 (2.2)	
GS upgrading at RP			0.089
Yes	186 (14.9)	194 (15.5)	
No	329 (26.3)	349 (27.9)	
Unknown	110 (8.8)	82 (6.6)	
PSA at RP			0.698
<10 ng/ml	465 (38.3)	483 (39.8)	
10–20 ng/ml	106 (8.7)	106 (8.7)	
>20 ng/ml	24 (1.9)	31 (2.6)	

RASi = renin-angiotensin inhibitor; GS = Gleason score; PSA = prostate-specific antigen; RP = radical prostatectomy.

### 3.2 Impact of RASi on histopathological characteristics

Comparison of GS data revealed no significant differences between the RASi and control groups in GS at biopsy (*p* = 0.172), GS at RP (*p* = 0.277), or GS upgrading from biopsy to RP (*p* = 0.089). There was also no difference in the incidence of positive resection margins (*p* = 0.109) or positive lymph nodes (*p* = 0.33) in the RP specimen (Table 2).

### 3.3 BCR after RP

**Fig. 2:**
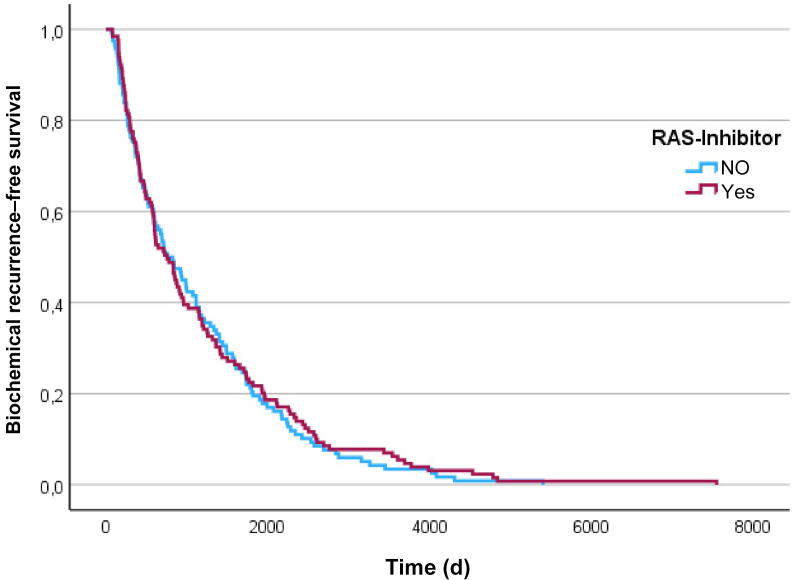
Kaplan-Meier curve on impact of RASi on BCR: There was no significant difference in BCR occurrence between the groups (*p* = 0.683). RAS = renin-angiotensin system; BCR = biochemical recurrence.

We evaluated differences in BCR during follow-up after RP. BCR was defined as an increase in PSA to > 0.2 ng/ml on two consecutive laboratory tests, in line with the European Association of Urology guidelines that applied at the time of data analysis [21]. Follow-up data on BCR occurrence were available for 1062 cases, of whom 541 were in the RASi group and 521 were in the RASi-naïve group. Median time to BCR was 757 d (mean 1202.17 d, range 72–5397) in the RASi group and 778 d (mean 1126.34 d, range 80–7540) in the RASi-naïve group, with no significant difference between the groups (*p* = 0.683). It is noteworthy that there were no significant differences in either the BCR rate (*p* = 0.258) or the median time to BCR (*p* = 0.683) between the groups (Table 3 and Fig. 2).

**Table 3:** BCR follow-up data for the two groups

Parameter	No RASi	RASi	*p* value
BCR, *n* (%)			0.258
Yes	150 (12)	149 (11.9)	
No	371 (29.7)	392 (31.4)	
Unknown	104 (8.3)	84 (6.7)	
Time to BCR (d)	778	757	0.683

RASi = renin-angiotensin inhibitor; BCR = biochemical recurrence (BCR).

## 4 Discussion

The impact of RASi intake on the occurrence and aggressiveness of various cancers and on treatment outcomes has been broadly discussed. RASi use in pancreatic cancer was associated with prolonged OS [22]. In ovarian cancer, RASi intake led to a notable decrease in collagen levels and enhanced perfusion in cancerous tissue, resulting in a reduction in tumor hypoxia [23]. Patients with non–small-cell lung cancer who were undergoing first-line platinum-based chemotherapy experienced longer median survival if they were receiving a concomitant RASi or AT1R blocker (11.7 vs 8.6 mo; hazard ratio [HR] 0.56; *p* = 0.03) [24].

Regarding PCa, various studies have yielded inconclusive findings. A meta-analysis by Song et al [14] revealed a significant reduction in the risk of cancer recurrence and mortality for patients on RASi therapy, with the reduction in risk ranging from 25% to 40%, depending on the type of cancer. Specific analysis for PCa also showed a benefit of RASi intake on disease-free survival, with a HR of 0.14 [14]. Another meta-analysis that included data from 55 studies corroborated these results and demonstrated a significant improvement in OS among cancer patients using RASi [15]. Furthermore, a systematic review and large meta-analysis of 20 000 patients from nine cohort studies suggested a decreased risk of PCa occurrence for patients receiving RASi treatment [16].

In contrast, a meta-analysis including data of more than 60 000 patients examined the impact of ARBs intake on the incidence of newly diagnosed cancers, and found that ARBs consumption was associated with a higher risk of PCa incidence [17]. This result is supported by two Finnish studies. A population-based case-control study involving 24 657 case-control pairs, revealed a marginal increase in the risk of PCa for patients using RASi in comparison to their RASi-naïve counterparts (odds ratio [OR] 1.16) [25]. Siltari et al [18] observed a slightly increased risk of developing PCa (HR 1.10) among 78 615 men using RASi for hypertension treatment.

To provide a comprehensive understanding of the impact of RASi on PCa, we compiled an overview of existing studies. Table 4 lists key findings from prior research on RASi in various settings related to PCa. Our review of the literature revealed that while multiple population-based studies have explored the effects of RASi, only one of these studies included a comparison arm. However, this particular study also included patients diagnosed with breast or colorectal cancer, thereby diluting the specificity for PCa.

**Table 4:** Overview of studies on the effects of RASi on prostate cancer prognosis

Study	PC stage	AHT types	Design	Patients	mFU	CC	Tx	Key results
Santala 2019 [26]	LPC	ACEi ARBs CCBs Diuretics BBs	RS	14 422	NA	No	RP	Lower risk of PC death with ARBs Higher risk for starting ADT with other AHTs
Cardwell 2014 [19]	HSPC	RASi (NS)	RS	1184	13 yr	No	NA	No increase in PCa mortality
Siltari 2020 [18]	HSPC	ACEi ARBs CCBs Diuretics BBs	RS	8253	7.6 yr	No	ADT	ARBs associated with better survival AHTs associated with higher PC risk
Uemura 2005 [27]	mCRPC	ARBs	PS	23	NA	No	PDAA	50% showed stable or improved PFS
Fiala 2024 [28]	mCRPC	ACEi ARBs CCBs Diuretics BBs	RS	300	29.3 mo	No	Enz/Abi Docetaxel Cabazitaxel ^223^Ra	Positive ACEi effect on OS in Enz/Abi group
Wilk 2021 [29]	mCRPC	RASi (NS)	RS	93	9.8 mo	No	Abi after failed CTx	Longer TTF for RASi users
Present study	LPC	RASi (NS)	RS	1250	25.9 mo	Yes	RP	No difference in BCR

ACEi = angiotensin-converting enzyme inhibitor; ADT = androgen deprivation therapy; AHT = antihypertensive agent; ARB = angiotensin II receptor blocker, BB = β-blocker; BCR = biochemical recurrence; CCB = calcium channel blocker; PC = prostate cancer; LPC = localized PC; HSPC = hormone-sensitive PC; mCRPC = metastatic castration-resistant PC; NS = not specified; RS = retrospective study; PS = prospective study; NA = not applicable; mFU = median follow-up; CC = comparison cohort; RP = radical prostatectomy; RASi = renin-angiotensin inhibitor; Tx= therapy; CTx = chemotherapy; PDAA = post-dexamethasone androgen ablation; Enz/Abi = enzalutamide/abiraterone; PFS = progression-free survival; OS = overall survival; TTF = time to treatment failure.

Due to the heterogeneity of results in the literature, our aim was to evaluate differences in PCa aggressiveness according to RASi use among patients who underwent RP for organ-confined PCa in one of four European centers between 1990 and 2023. We analyzed multiple parameters related to the aggressiveness of local PCa and recurrence among RASi users and their RASi-naïve counterparts in a large, well-balanced cohort of 1250 men.

In comparison to the studies listed in Table 4, the present study is distinguished by the inclusion a comparison arm and an exclusive focus on PCa patients, which facilitates a more direct assessment of RASi effects in this specific population. Moreover, the substantial size of our study cohort enhances the robustness and reliability of our findings.

Regarding baseline parameters, patients in the RASi group had higher median BMI (*p* < 0.001), which is not surprising, as the association between obesity and hypertension has been extensively studied for several decades. However, there were no significant differences in other key parameters between the two groups. Specifically, the rate of GS upgrading from biopsy to RP (*p* = 0.089), the initial PSA level (*p* = 0.698), and the rate of negative resection margins (*p* = 0.109) were comparable between the two groups. The absence of significant differences in parameters other than BMI suggests that while RASi therapy may influence certain physiological parameters, based on its systemic effects, its role in modulating local tumor aggressiveness or altering the course of PCa in the context of surgical outcomes remains unclear. Without additional correlations, such as lymph node involvement, our data do not support the hypothesis that RASi could significantly impede tumor cell migration or metastasis in PCa. This underscores the need for further research to clarify the potential impacts of RASi on the tumor microenvironment and cancer progression.

Our findings are in line with several studies that suggested that there is neither an increase nor a decrease in the risk of PCa occurrence, progression, or mortality. A large population-based study of 5849 PCa patients in the UK revealed no significant difference in cancer-specific mortality for patients using RASi (OR 0.78) [19]. In addition, a Finnish cohort study reported a survival benefit only for patients using ARBs, while no significant effect of RASi on PCa-related mortality was observed [26].

Furthermore, analysis ofour large cohort did not show a significant difference in BCR incidence (*p* = 0.258) or the time to BRC (*p* = 0.683) between the two groups. Therefore, we can postulate that RASi medication in patients with localized PCa had no impact on the BRC rate or recurrence-free survival time in our study population.

Our study has several important limitations that need to be considered when interpreting the results. Due to the retrospective nature of the study, access to complete follow-up data was limited. For example, recurrence data for patients who received postoperative follow-up from health care providers outside the participating hospitals were not fully captured. Consequently, while follow-up information was available for 1062 patients (84.96%), the date of BCR was only available for 285 patients (22.8%). The restricted follow-up information could introduce bias, as patients with available follow-up data may not be fully representative of the entire cohort. Consequently, this limitation affects our ability to draw robust conclusions regarding long-term outcomes, including BRC rates. Secondly, the exact date of RASi initiation is unknown for our patient cohort, as antihypertensive therapy is usually prescribed by the primary care physician. However, considering that the median age of our RASi group is 64 yr, and given that RASi is typically prescribed for long-term management of hypertension, we expect that the treatment duration is likely to be substantial. This assumption is based on the common medical practice of prescribing RASi to patients within this age group for prolonged periods. In addition, the absence of detailed data on treatment duration reflects the real-world setting of the study, in which tracking of medication history over long periods can be challenging, particularly when multiple health care providers are involved. This missing information is representative of the gaps commonly encountered in retrospective analyses and highlights the importance of considering real-world data limitations when interpreting study results. Nonetheless, we recognize the importance of this information and that without precise information on RASi therapy duration, it is difficult to establish a clear relationship between RASi use and PCa aggressiveness.

These limitations underscore the need for cautious interpretation of our findings and highlight the importance of further research with more comprehensive follow-up data and precise treatment timelines to better understand the impact of RASi on PCa aggressiveness and recurrence.

## 5 Conclusions

In conclusion, our comprehensive analysis of 1250 PCa patients found no significant differences in clinical outcomes between those receiving RASi therapy and their RASi-naïve counterparts. Furthermore, there was no significant difference in BCR incidence between the two groups. Our study highlights the complexity of interpreting the influence of RASi on PCa aggressiveness and underscores the need for further research to conclusively determine the impact of RASi use in the clinical course of PCa.

  ***Author contributions***: Isabel Heidegger had full access to all the data in the study and takes responsibility for the integrity of the data and the accuracy of the data analysis.

  *Study concept and design*: Artamonova, Kafka, Heidegger.

*Acquisition of data*: All authors.

*Analysis and interpretation of data*: Artamonova, Kafka, Heidegger.

*Drafting of the manuscript*: Artamonova, Kafka, Heidegger.

*Critical revision of the manuscript for important intellectual content*: All authors.

*Statistical analysis*: Steiner.

*Obtaining funding*: None.

*Administrative, technical, or material support*: None.

*Supervision*: Heidegger.

*Other*: None.

  ***Financial disclosures:*** Isabel Heidegger certifies that all conflicts of interest, including specific financial interests and relationships and affiliations relevant to the subject matter or materials discussed in the manuscript (eg, employment/affiliation, grants or funding, consultancies, honoraria, stock ownership or options, expert testimony, royalties, or patents filed, received, or pending), are the following: None.

  ***Funding/Support and role of the sponsor*:** None.

  ***Ethical considerations*:** This study was performed in accordance with local ethics standards.
